# MLAA-based attenuation correction of flexible hardware components in hybrid PET/MR imaging

**DOI:** 10.1186/s40658-017-0177-4

**Published:** 2017-03-01

**Authors:** Thorsten Heußer, Christopher M. Rank, Yannick Berker, Martin T. Freitag, Marc Kachelrieß

**Affiliations:** 1 Medical Physics in Radiology, German Cancer Research Center (DKFZ), Im Neuenheimer Feld 280, Heidelberg, 69120 Germany; 2 Department of Radiology, University of Pennsylvania, 3620 Hamilton Walk, Philadelphia, 19104 PA USA; 3 Physics of Molecular Imaging Systems, RWTH Aachen University, Pauwelsstraße 19, Aachen, 52074 Germany; 4 Department of Radiology, German Cancer Research Center (DKFZ), Neuenheimer Feld 280, Heidelberg, 69120 Germany

**Keywords:** PET/MR, Attenuation correction, Hardware attenuation, MLAA

## Abstract

**Background:**

Accurate PET quantification demands attenuation correction (AC) for both patient and hardware attenuation of the 511 keV annihilation photons. In hybrid PET/MR imaging, AC for stationary hardware components such as patient table and MR head coil is straightforward, employing CT-derived attenuation templates. AC for flexible hardware components such as MR-safe headphones and MR radiofrequency (RF) surface coils is more challenging. Registration-based approaches, aligning CT-based attenuation templates with the current patient position, have been proposed but are not used in clinical routine. Ignoring headphone or RF coil attenuation has been shown to result in regional activity underestimation values of up to 18%.

We propose to employ the maximum-likelihood reconstruction of attenuation and activity (MLAA) algorithm to estimate the attenuation of flexible hardware components. Starting with an initial attenuation map not including flexible hardware components, the attenuation update of MLAA is applied outside the body outline only, allowing to estimate hardware attenuation without modifying the patient attenuation map. Appropriate prior expectations on the attenuation coefficients are incorporated into MLAA. The proposed method is investigated for non-TOF PET phantom and ^18^F-FDG patient data acquired with a clinical PET/MR device, using headphones or RF surface coils as flexible hardware components.

**Results:**

Although MLAA cannot recover the exact physical shape of the hardware attenuation maps, the overall attenuation of the hardware components is accurately estimated. Therefore, the proposed algorithm significantly improves PET quantification. Using the phantom data, local activity underestimation when neglecting hardware attenuation was reduced from up to 25% to less than 3% under- or overestimation as compared to reference scans without hardware present or to CT-derived AC. For the patient data, we found an average activity underestimation of 7.9% evaluated in the full brain and of 6.1% for the abdominal region comparing the uncorrected case with MLAA.

**Conclusions:**

MLAA is able to provide accurate estimations of the attenuation of flexible hardware components and can therefore be used to significantly improve PET quantification. The proposed approach can be readily incorporated into clinical workflow.

## Background

Accurate quantification in positron emission tomography (PET) mandates attenuation correction (AC), which compensates for the effect of photon attenuation. AC is a major challenge in combined PET/MR imaging since the available magnetic resonance (MR) information cannot be directly converted into corresponding PET attenuation coefficients [1–3]. The standard approach for MR-based AC (MRAC) employed in clinical routine is to acquire a series of dedicated MR images, followed by segmentation into three to four tissue classes, which are then assigned pre-defined attenuation coefficients [4–6]. Since bone is usually not accounted for in these methods, MRAC results in an underestimation of the reconstructed PET activity [7–9]. Ongoing efforts aim at improving MRAC by employing dedicated MR sequences, e.g., ultrashort-echo-time (UTE) sequences [10–12], or by making use of atlas-based methods [13, 14]. Another approach for AC makes use of the fact that the PET emission data contain information about both the activity and the attenuation distribution [15]. Emission-based AC exploits this fact, simultaneously reconstructing activity and attenuation distributions from either time-of-flight (TOF) [16–19] or non-TOF [20] PET emission data.

In hybrid PET/MR imaging, attenuation of the 511 keV annihilation photons is caused both by the patient and by system hardware placed within the PET field-of-view (FOV). Theses hardware components (e.g., patient table, various MR coils, pneumatic headphones,...) are not visible with standard MR sequences employed in clinical routine [21–25]. Hardware components are thus usually optimized for PET transparency, i.e., they are designed such that they attenuate the 511 keV photons as little as possible [26]. Despite these efforts to minimize the total attenuation of individual hardware components, ignoring hardware-induced attenuation results in both qualitative and quantitative errors in the reconstructed PET images, demanding AC [21–25, 27–35].

For stationary components, such as patient table and integrated signal receiving MR radiofrequency (RF) coils (e.g., head/neck, spine, and breast coils), AC is relatively straightforward. A transmission scan using computed tomography (CT) or rotating rod sources is performed to obtain an attenuation template of the corresponding hardware component [21–24, 30–32]. This template is then converted to 511 keV [36–38] and incorporated into the patient attenuation map at a fixed position. Since position and shape of stationary hardware components do not differ between scans, the attenuation templates need to be created only once. This is usually taken care of by the vendors and the relevant attenuation templates are automatically selected and incorporated into the attenuation map in clinical routine [26].

AC for flexible hardware components, such as flexible RF surface coils, MR-safe pneumatic headphones, and positioning aids, is more challenging [23–25, 27–29, 33–35]. In contrast to stationary components, flexible hardware components differ, in general, in their spatial position and in their shape between scans. Thus, attenuation maps of flexible hardware components need to be scan-specific. To obtain such scan-specific attenuation maps, registration-based methods have been proposed, suggesting to incorporate pre-acquired attenuation templates of the corresponding hardware components into the vendor-provided attenuation maps. These methods require performing a transmission scan (CT or rotating rod sources) of the corresponding hardware and scaling to 511 keV. Non-rigid registration is then employed to accurately align the scaled attenuation template using either bi-modal fiducial markers [22, 25, 27, 28, 33] or dedicated MR sequences (e.g., UTE), which are capable of retrieving some signal of the given hardware component [25, 29, 35, 39]. In current clinical practice, however, attenuation of flexible hardware components is neglected. This results in an underestimation of regional activity values of up to 18% for torso RF surface coils [23–25, 27, 38], and up to 16% for MR-safe pneumatic headphones [22, 33, 34].

To improve PET quantification in hybrid PET/MR imaging, we propose a method to estimate the attenuation of flexible hardware components employing the maximum-likelihood reconstruction of attenuation and activity (MLAA) algorithm [15]. The proposed approach simultaneously reconstructs activity and attenuation distributions from the PET emission data. In this work, the attenuation distribution is estimated only outside the patient body outline, i.e., the patient attenuation map is considered to be known and not modified during reconstruction. In contrast to all other methods proposed for AC of flexible hardware components [22, 25, 27–29, 33, 35], our method does not rely on the co-registration of pre-acquired attenuation templates of the corresponding hardware but estimates the attenuation directly from the PET emission data. A similar approach has been proposed to estimate missing parts of the patient attenuation map in case of truncation of the MR data used for MRAC [40]. In addition to testing their emission-based patient attenuation map completion on several clinical data sets, Nuyts et al. also performed a simulation experiment, demonstrating that MLAA is in principle capable of providing attenuation information of hardware components placed within the PET FOV.

In this work, a simulation study was performed to investigate the differences between emission-based truncation completion and emission-based hardware attenuation estimation. Moreover, we evaluated MLAA-based hardware attenuation estimation for pneumatic headphones and for a six-channel torso RF surface coil using both phantom and patient non-TOF PET data acquired with a Siemens Biograph mMR (Siemens Healthineers, Erlangen, Germany).

## Methods

### Algorithm

**Fig. 1:**
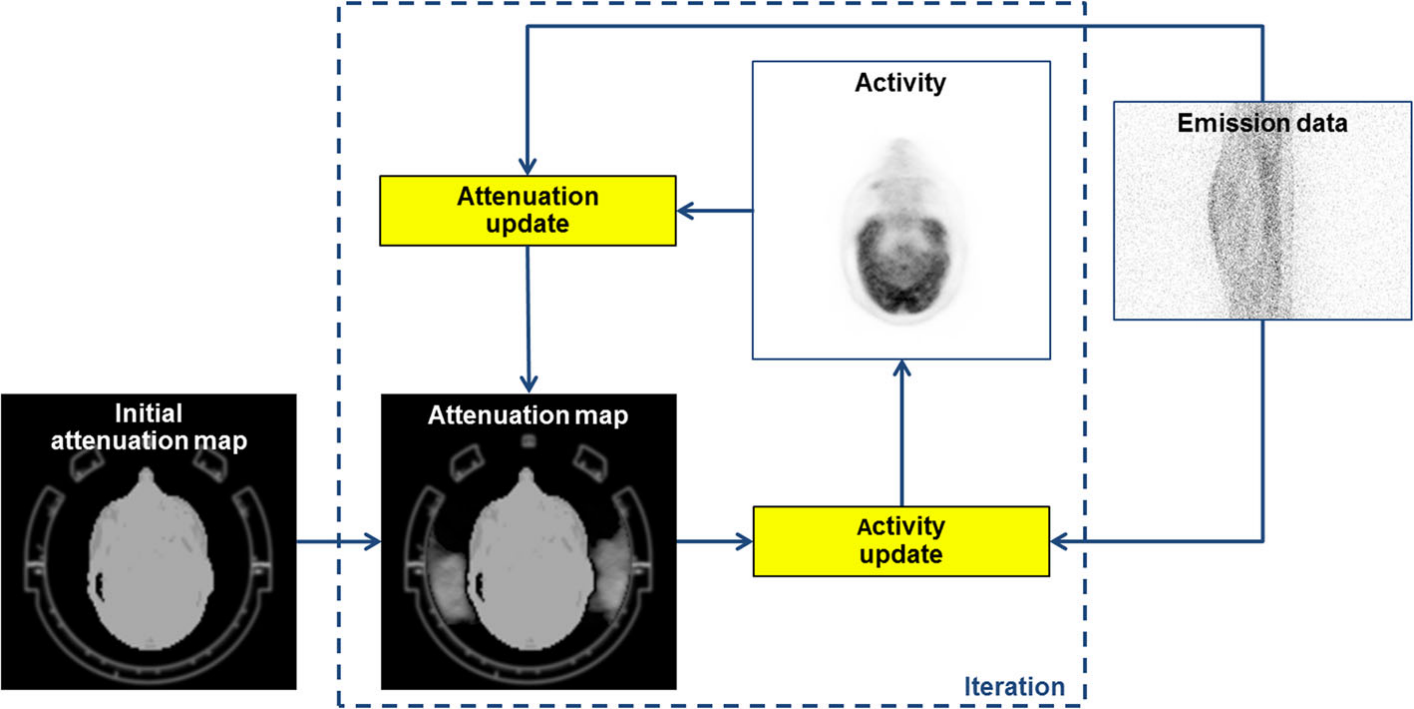
Workflow of the xMLAA algorithm for estimation of hardware attenuation. Input data are the PET emission data and an initial attenuation map of the phantom/patient. The initial attenuation map does not include information on the attenuation of flexible hardware components. Activity and attenuation distributions are updated in an alternating manner. The attenuation update is only applied outside the patient outline

A modified MLAA algorithm [15] is used to simultaneously reconstruct activity and attenuation distributions from the PET emission data. The general workflow is depicted in Fig. 1. Input data are the PET emission data and an initial attenuation map of the investigated patient, which is either the standard MR-based attenuation map or, e.g., derived from CT using atlas-based methods. In both cases, the initial attenuation map includes information about stationary hardware components such as the patient table and the MR head coils. However, information about flexible hardware components is not included in the initial attenuation map. The proposed approach is iterative, updating attenuation and activity distribution in an alternating manner. The attenuation update is only performed outside the patient body outline without modifying the patient attenuation map. Therefore, to differentiate the MLAA-based hardware attenuation estimation approach from the original MLAA algorithm for patient attenuation estimation, our proposed algorithm will be referred to as external MLAA (xMLAA) in the following.

#### Objective function

The algorithm aims at reconstructing the activity distribution ***λ***=(*λ*_1_,…,*λ*_*I*_)^T^ and the attenuation distribution ***μ***=(*μ*_1_,…,*μ*_*I*_)^T^ from the measured PET emission data ***p***=(*p*_1_,⋯,*p*_*J*_)^T^, where *I* gives the number of image voxels and *J* gives the total number of lines of response (LORs). The algorithm seeks to optimize the objective function

1 $$ Q({\boldsymbol\lambda}, {\boldsymbol\mu}) = L({\boldsymbol\lambda}, {\boldsymbol\mu})+\beta_{\mathrm{S}}L_{\mathrm{S}}({\boldsymbol\mu})+\beta_{\mathrm{I}}L_{\mathrm{I}}({\boldsymbol\mu}),   $$

where *L*(***λ***,***μ***) is the standard log-likelihood function given as

2 $$ L({\boldsymbol\lambda}, {\boldsymbol\mu}) = \sum_{j}{(p_{j}\ln{\hat{p}_{j}}-\hat{p}_{j})},   $$

and

3 $$ \hat{p}_{j} = \frac{1}{a_{j} n_{j}}\sum_{i}{M_{ij}\lambda_{i}}+\frac{s_{j}}{n_{j}}+r_{j}.   $$

The values $\hat {p}_{j}$ are the expected number of coincidences along a given LOR *j*, i.e., the estimated emission data. *M*_*ij*_ are the elements of the system matrix, and *n*_*j*_, *s*_*j*_, and *r*_*j*_ represent the normalization, scatter, and randoms contribution to LOR *j*, respectively. The attenuation correction factor (ACF) for LOR *j* is given by

4 $$ a_{j} = \exp{\left(\sum_{i}{\mu_{i}}l_{ij}\right)},   $$

where *l*_*ij*_ denotes the intersection length of voxel *i* with LOR *j*.

The objective function (1) includes two prior terms, *L*_S_(***μ***) and *L*_I_(***μ***), which can be weighted relative to the likelihood function *L*(***μ***) using the parameters *β*_S_ and *β*_I_, respectively. The smoothing prior *L*_S_(***μ***) is realized as the logarithm of a Gibbs probability distribution with the Geman-McClure function as potential function [41–43]. It thus penalizes attenuation distributions ***μ*** which are not locally smooth. The intensity prior *L*_I_(***μ***) favors the occurrence of pre-defined attenuation coefficients, e.g., for air and for the hardware material. Deviations from these pre-selected values are penalized. It is realized as a bi-modal Gaussian-like probability distribution defined by the mean values *μ*_air_ and *μ*_hardware_ and their corresponding standard deviations *σ*_air_ and *σ*_hardware_, respectively. The choice of the mean values and their distribution is explained further below. Since the attenuation update introduced further below only requires the gradient of the logarithm of the probability distribution, this gradient is directly defined using piecewise linear functions. More details on the prior terms are given in references [15] and [20].

#### Update equations

Optimizing the objective function (1) keeping ***μ*** constant yields the ordinary-Poisson maximum likelihood expectation maximization (OP-MLEM) activity update [44]

5 $$ \lambda_{i}^{(u+1)} = \lambda_{i}^{(u)} \frac{1}{\sum_{j}{M_{ij}/(a_{j}^{(u)}n_{j})}} \sum_{j}{M_{ij} \frac{p_{j}}{\sum_{k}{M_{kj}\lambda_{k}^{(u)}+a_{j}^{(u)}(s_{j}+r_{j}n_{j})}}},   $$

where *u* gives the update index. The attenuation update is obtained by optimizing (1) for fixed ***λ*** and using a gradient-descent method for transmission tomography [45, 46]:

6 $$\begin{array}{*{20}l} &\mu_{i}^{(u+1)} = \mu_{i}^{(u)} \\ &+ \alpha \frac{\sum_{j}{\left(l_{ij}(\hat{p}_{j}^{(u)} - p_{j})\frac{\hat{p}_{j}^{(u)}-s_{j}/n_{j}-r_{j}}{\hat{p}_{j}^{(u)}}\right)} + \frac{\partial}{\partial \mu_{i}}(L_{\mathrm{S}}+L_{\mathrm{I}})} {\sum_{j}{\left(l_{ij} (\hat{p}_{j}^{(u)}-s_{j}/n_{j}-r_{j}) \left(1-\frac{p_{j}(s_{j}/n_{j}+r_{j})}{\hat{p}_{j}^{(u)2}}\right) \sum_{k}{l_{kj}}\right)} - \sum_{k}{\frac{\partial^{2}}{\partial \mu_{i} \partial \mu_{k}}(L_{\mathrm{S}}+ L_{\mathrm{I}})}}.  \end{array} $$

Here, *α* is a relaxation parameter. The estimation of the emission data $\hat {p}_{j}^{(u)}$ is given by Eq. (3) using the current attenuation ***μ*** ^(*u*)^ and activity ***λ*** ^(*u*+1)^ distributions (the activity is updated first, hence it corresponds to update index *u*+1). After each attenuation update, negative attenuation coefficients are avoided by truncating corresponding values to zero. The attenuation update is restricted to a region outside the patient body outline, which is assumed to contain the hardware components. This is explained in more detail further below. In contrast, the activity update is applied within the entire PET FOV.

### Simulations

The major difference between emission-based attenuation map completion, as suggested in reference [40], and emission-based hardware attenuation estimation, as proposed in this work, is that in the former case, the missing anatomy has non-zero tracer uptake (“warm objects”) while in the latter case, the hardware components contain zero activity (“cold objects”). To demonstrate the different behavior of xMLAA-based attenuation estimation in case of warm and cold objects, a simulation study was performed. A voxel phantom was simulated, comprised of a main body (15 cm diameter cylinder, *μ*=0.01 mm^−1^, *λ*=1 a.u.) with two ellipsoidal objects to each side (*μ*=0.01 mm^−1^), which roughly model the earpads of the MR-safe pneumatic headphones introduced below. In this simulation study, one of the earpads was assigned non-zero activity (*λ*=1 a.u.), while the other earpad was not assigned any activity. We simulated noiseless PET emission data based on the activity distribution and considering attenuation due to the main body and the earpads. Randoms and scatter were not simulated.

### Experiments

In this work, we investigated the proposed algorithm performing phantom measurements and evaluating clinical patient data. All PET data were acquired with an integrated clinical PET/MR device (3T Biograph mMR, Siemens Healthineers, Erlangen, Germany) [47]. CT-based attenuation maps of the phantoms and the hardware components were obtained with a clinical spiral CT device (SOMATOM Definition Flash, Siemens Healthineers, Forchheim, Germany)

#### Hardware components

**Fig. 2:**
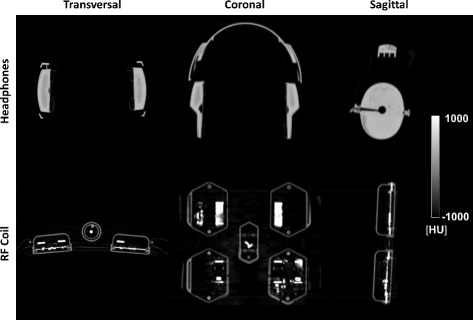
CT images of headphones and RF surface coil. The headphones are composed of two earpads connected with a flexible headband. The RF coil contains highly attenuating materials with CT values larger than 3071 HU, mandating the use of an extended CT scale. CT grayscale windowing: *C*=0 HU,*W*=2000 HU

The hardware components investigated in this study were a pair of pneumatic MR-safe headphones [33, 34] and a six-channel torso RF matrix coil [25, 27, 38], which were shipped with the Biograph mMR. Both components are routinely used in clinical workflow but are not considered during AC. Two CT scans showing the headphones (HP) and the RF surface coil are presented in Fig. 2.

#### Phantom measurements

Phantom measurements were performed since for these, both a reference scan without hardware attached and a CT-based attenuation template including hardware could be acquired. This allowed for a quantitative evaluation of the proposed xMLAA-based approach in comparison to either the reference or the CT-based template. For all phantom measurements described in detail below, we acquired PET data without and with the hardware components placed in the PET FOV. The data acquired without hardware components serve as reference. An overview of the phantom experiments is given in Table 1. For all phantom measurements, PET data were acquired for a single bed position only. During each measurement, we acquired 50×10^6^ prompt events after randoms correction. We used CT-derived attenuation maps of the phantoms since the plastic housing of the phantoms is not visible in MR-based attenuation maps.

**Table 1:** Characteristics of the phantom experiments and patient measurements. The acquired counts are stated prior to randoms correction and for scans with hardware components attached. HP = headphones, RF = RF surface coil

	Phantom /	Activity	Acquired	Reference	CT data
	bed position	[MBq]	counts ×10^6^	available	available
HP experiment	Cylinder phantom	48 (^68^Ga)	59	Yes	Yes
RF experiment	Pelvis phantom	55 (^68^Ga)	62	Yes	Yes
HP patients	Head/neck	228±13	62±19	No	No
	(*n*=6)	(^18^F-FDG)			
RF patients	Abdomen	236±9	64±6	No	No
	(*n*=5)	(^18^F-FDG)			

**Fig. 3:**
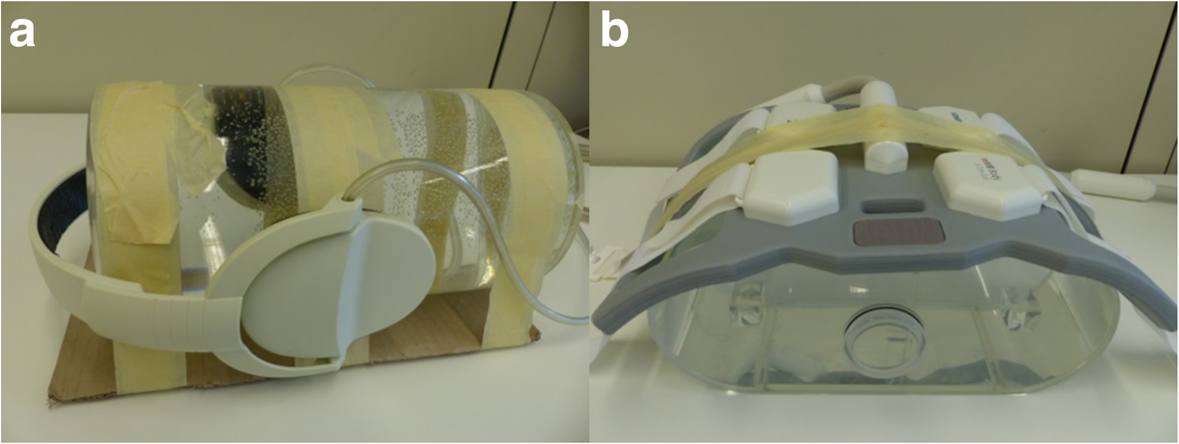
Photos of the phantoms with hardware components attached. **a** The positioning of the headphones in the HP experiment corresponds to the set-up as present in patient examinations. The headphones were additionally fixed to the cylinder phantom using adhesive tape (not shown in the picture). **b** For the RF experiment, the RF coil was placed on top of the pelvis phantom and fixed with adhesive tape

HP experiment: we used a 15 cm diameter cylinder phantom made of PMMA that fits into the MR head coil. The phantom was filled with deionized water and 0.9*%* NaCl, improving MR homogeneity compared to pure water [48]. The phantom was placed on a thin cardboard for stabilization and securely fixed with adhesive tape. Then, 48 MBq of ^68^Ga were administered. We placed the phantom inside the head coil, which was fixed in the appropriate position. A PET scan without headphones attached was performed (reference scan). The phantom was then removed from the head coil and the headphones were attached and fixed with adhesive tape as seen in part (a) of Fig. 3. With the headphones attached, the phantom was placed in identical position and a second PET scan was performed. Decay correction was performed to account for tracer decay during and between the scans. After the activity had decayed, a CT scan of the phantom with the headphones still in identical position was performed the next day (CT imaging parameters: 140 kVp, 700 mAs, 500×500 mm^2^ FOV, 512×512 image matrix, 2 mm slice thickness). We used bilinear scaling [37] and an in-house affine registration algorithm aligning the scaled CT image with the MR-based attenuation map to obtain a CT-based attenuation map of the phantom including the headphones. To remove streak artifacts and background noise, all attenuation coefficients in the scaled CT image below *μ*=0.001 mm^−1^ were set to zero. The CT patient table was removed manually. A CT-based attenuation map of the cylinder phantom without headphones was obtained by manually segmenting and removing the headphones.

RF experiment: The RF experiment followed the procedure of the HP experiment. We used a dedicated pelvis phantom [49], which, for the work presented here, consisted of a PMMA housing filled with 11 L deionized water and 0.9*%* NaCl. 55 MBq of ^68^Ga were administered. We then placed the phantom on the PET/MR patient table and performed a reference PET scan without RF surface coil attached. For the second scan, we fixed an RF surface coil on top of the phantom using adhesive tape, as seen in part (b) of Fig. 3, without moving the phantom. Decay correction was performed to account for tracer decay during and between the scans. CT scans with the RF coil still in identical position and without RF coil were performed the next day, after the activity had decayed. CT imaging parameters were identical to the ones used in the HP experiment. Additionally, the extended CT scale was switched on during reconstruction, since the RF coil contains highly attenuating components with CT-values larger than 3071 HU. We thus applied bilinear scaling using the parameters suggested in reference [38], which have been shown to improve CT-based AC of highly attenuating hardware materials. Affine registration was used to align the scaled CT images with and without RF coil with the MR-based attenuation map. As in the HP experiment, CT-based streak artifacts and background noise were removed by setting all attenuation coefficients in the scaled CT image below *μ*=0.001 mm^−1^ to zero.

Effect of MRAC-based errors: In the previously described HP and RF experiments, we used CT-derived attenuation maps of the phantoms. To evaluate the effect of inaccurate attenuation maps on hardware attenuation estimation employing xMLAA, typical MRAC-based errors were simulated, modifying the CT-based attenuation maps. In case of the HP experiment, a spherical region (3 cm diameter) was added in vicinity of one of the earpads and assigned zero attenuation, thus simulating an extensive air cavity. For the RF experiment, a fat-water tissue inversion was simulated by scaling the CT-derived attenuation coefficients corresponding to the water-filled pelvis phantom with a factor of 0.85. Note, it was not the aim of this work to evaluate the direct impact of such MRAC-based errors on PET quantification and image quality, which has been presented in detail elsewhere [7–9, 50, 51]. Here, we were interested in the effect of these errors on xMLAA-based hardware attenuation estimation and the corresponding, indirect impact on PET quantification.

Effect of inaccurate scatter estimates: Scatter estimation requires knowledge about the attenuation distribution. In all phantom experiments and patient data sets presented in this work, scatter estimation is based on the uncorrected attenuation map, i.e., without hardware components present, as this corresponds to the clinical setting where hardware attenuation is neglected. However, since the annihilation photons may also be scattered by the hardware, the scatter estimate based on attenuation maps not including the hardware components may be inaccurate. Therefore, to investigate the effect of such inaccurate scatter estimates and the potential benefit when scatter estimation is based on attenuation maps including hardware components, we calculated and compared estimated scatter distributions corresponding to the uncorrected, xMLAA-, and CTAC-based attenuation maps. The impact of the different scatter estimates on PET quantification was also evaluated.

#### Patient data

To demonstrate the feasibility of the proposed approach for clinical data, we also investigated several patient data sets with either headphones or RF coils in the PET FOV. Patient data were retrospectively collected from a study approved by the local ethics committee. We investigated PET data from six patients wearing headphones (HP patients) and five patients with RF surface coils attached (RF patients). All patients were administered ^18^F-FDG. An overview of the patient data is given in Table 1. Other than for the phantom data, neither a reference scan nor aligned CT-based templates of the hardware components were available. We used PET data of single bed positions only, corresponding to the head/neck region (HP patients) or the abdominal region (RF patients), respectively. The HP patients were placed within the head coil. In case of the RF patients, 3 to 4 partially overlapping RF coils were used to cover the entire upper part of the body. Consequently, for the single bed position corresponding to the abdominal region that was investigated here, parts of two different RF coils could be present. For patient AC, the vendor-provided MR-derived attenuation maps [4] were used. These MRAC attenuation maps include air, lung, fat, and soft tissue attenuation coefficients. Bone attenuation is not considered, but treated as soft tissue. Moreover, due to the limited MR FOV, the patient attenuation maps may suffer from truncation.

#### Data processing

**Fig. 4:**
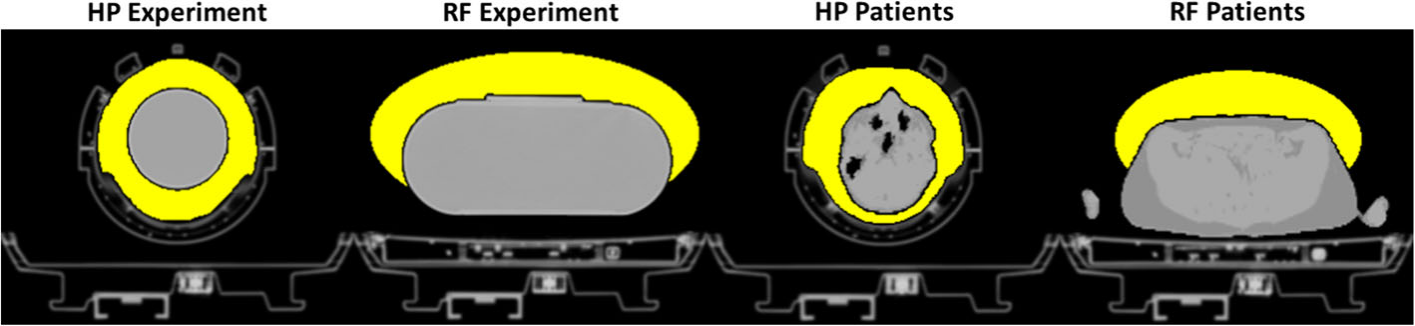
Initial attenuation maps and hardware masks. Shown here are the initial CT- or MR-based phantom or patient attenuation maps including the vendor-provided attenuation of stationary hardware components (head coil and patient table) and the corresponding hardware masks (*yellow*). The hardware mask defines the region where the xMLAA attenuation update is applied to estimate the attenuation of flexible hardware components (headphones or RF coil)

We performed xMLAA for hardware attenuation estimation for those scans where either headphones or the RF surface coil where located in the PET FOV. Estimates for normalization, randoms, and scatter were obtained using the Siemens e7tools offline reconstruction software (version VA20, Siemens Healthineers, Erlangen, Germany). xMLAA was initialized with CT-based attenuation maps in case of the phantom experiments and with MR-based attenuation maps in case of the patient data. In both cases, the initial attenuation maps did not contain flexible hardware attenuation information (headphones, RF coil). Scatter estimation obtained employing the single scatter simulation (SSS) [52] implemented in the e7tools software was thus based on imperfect attenuation maps. Attenuation of stationary hardware components (patient table, head coil) was included as provided by the vendor. The initial attenuation maps for the different phantom experiments and for representative patient data sets are shown in Fig. 4.

As mentioned previously, the xMLAA attenuation update is only applied outside the phantom or patient outline, hence the “x” for “external”. The region where xMLAA is applied, i.e., where flexible hardware components are assumed to be located, is referred to as hardware mask. Hardware masks for the different phantom experiments and for representative patient data are illustrated in Fig. 4. In those cases where the head coil poses a physical boundary for the possible location of the headphones (HP experiment and HP patients), the hardware mask was set to the manually segmented interior of the head coil. In case of the RF experiment and the RF patients, no such physical boundary was present. We rather chose the RF hardware mask to be of elliptical shape within the transversal plane, enclosing the true physical outline of the RF coil. Figure 4 only shows the transversal shape of the hardware masks, i.e., for a given *z*-plane. In axial direction, i.e., along the scanner axis, the hardware masks are restricted to *z*-planes which correspond to central parts of the PET detector. Specifically, the outermost 15 *z*-planes to both sides of the PET detector (127 *z*-planes in total) are not included in the hardware mask. In other words, the xMLAA attenuation update is only performed for central *z*-planes. This is because, using PET data from single bed positions only, the sensitivity in outer *z*-planes is very low such that emission-based attenuation estimation results in very high noise levels. The “background trick”, introduced in reference [15] and enforcing zero attenuation for LORs with no measured counts, cannot be applied, since it would also suppress non-zero attenuation of cold objects, such as flexible hardware components.

The xMLAA algorithm employed here is an in-house implementation based on a Joseph-type single beam forward and backprojection [53]. Each of the 50 xMLAA iterations was comprised of one activity and one attenuation update, each of which made use of all available LORs, i.e., no subsets were used for xMLAA. The relaxation parameter used in the attenuation update (6) was set to *α*=4.0. The weighting parameters, which are crucial for the outcome of xMLAA, were empirically derived and set to *β*_S_=5.0 and *β*_I_=0.01, respectively. Pre-defined attenuation values and their standard deviations as used by the intensity prior were *μ*_air_=0.0±0.0001 mm^−1^ and *μ*_hardware_=0.01±0.0020 mm^−1^. The main task of the intensity prior was to suppress non-zero attenuation in the background, i.e., in regions where only air was expected. Hence, a small standard deviation *σ*_air_=0.0001 mm^−1^ was chosen for the air expectation. However, if no other pre-defined, expected attenuation coefficient was incorporated into the attenuation update, hardware attenuation was greatly underestimated. Therefore, an additional hardware mode was added, modeling the expectations on the hardware attenuation coefficients. Compared to the air expectations, a much wider standard deviation *σ*_hardware_=0.0020 mm^−1^ was chosen, allowing for a wide range of hardware attenuation coefficients.

Final reconstructions were performed using the e7tools. We used ordered subset expectation maximization (OSEM) with 3 iterations and 21 subsets. Reconstruction results were smoothed using a 3D Gaussian filter with FWHM=5 mm. Volume dimensions were 344×344×127 with a voxel size of 2.09 ×2.09 ×2.03 mm for all data sets. We performed reconstructions using the CT- or MR-based attenuation map without hardware (uncorrected), the xMLAA-based attenuation map, and a CT-based attenuation map including hardware, if available. For the phantom data, a reference reconstruction was performed for the data corresponding to the scan without hardware present.

## Results

### Simulations

**Fig. 5:**
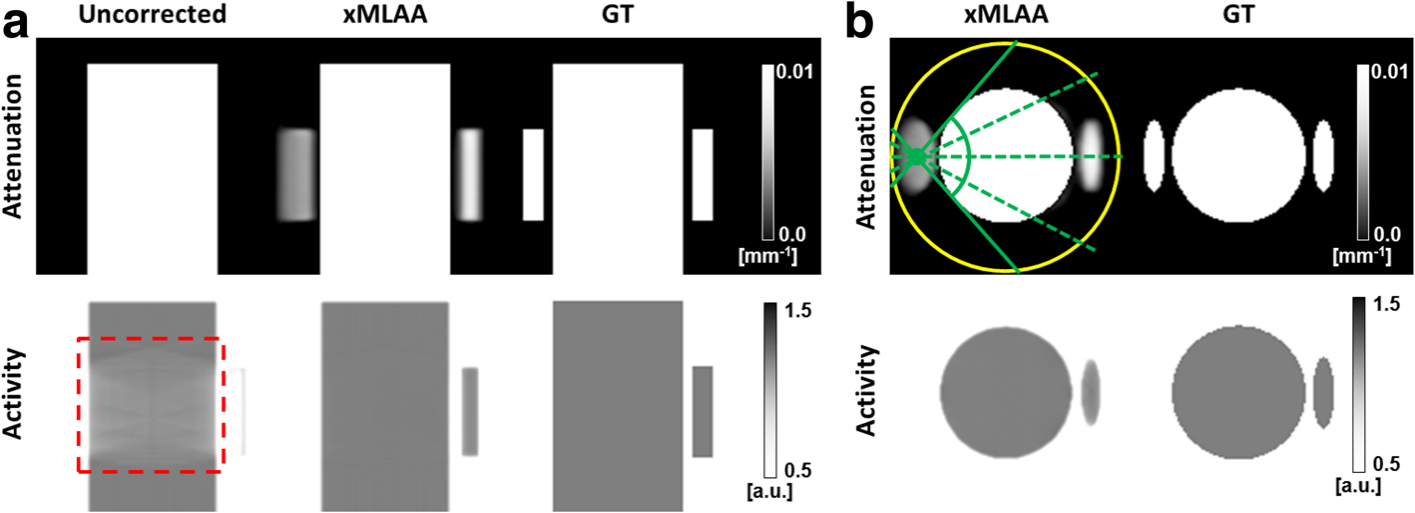
Results for the simulation study. **a** Coronal views of the uncorrected, xMLAA-based, and ground truth (GT) attenuation maps and corresponding activity distributions. The *red box* indicates the 3D region used during evaluation. **b** Transversal views of the xMLAA-based and ground truth (GT) attenuation maps and corresponding activity distributions. The *yellow circle* illustrates the outer boundary of the hardware mask. For the point indicated by the *green dot* and located within the cold object, only LORs which lie between the *solid green lines* contain attenuation information. This is the case for, e.g., the three LORs represented by the *dashed lines*

The results of the simulation experiment are presented in Fig. 5. In part (a), coronal views of the uncorrected, xMLAA-based, and ground truth (GT) attenuation maps and corresponding activity distributions are shown. In the uncorrected case, i.e., neglecting the attenuation of both the warm and the cold object, severe activity underestimation is present, especially for the region in between the warm and cold objects. The average activity error within the 3D region indicated by the red box in Fig. 5 is −5.8*%* compared to the ground truth. xMLAA can recover the attenuation of both the warm and the cold object and thus compensate for the activity underestimation, reducing the average activity error to below +0.1*%*. However, the shape of the cold object cannot be accurately reconstructed and it appears to be too wide in comparison to the ground truth. The reason for this is illustrated in part (b) of Fig. 5. Only LORs which cross both the cold object and the main body, which has non-zero tracer uptake, contain information on the attenuation of the cold object. For the point defined by the green dot, these are all LORs which lie between the solid green lines, e.g., the LORs represented by the three dashed lines. All other LORs crossing the cold object do not contain any emission data and thus no information on the attenuation. This is in contrast to the emission-based attenuation estimation of warm objects, as in reference [40], where all LORs crossing the object do contain emission data and thus attenuation information.

### Phantom data

#### HP experiment

**Fig. 6:**
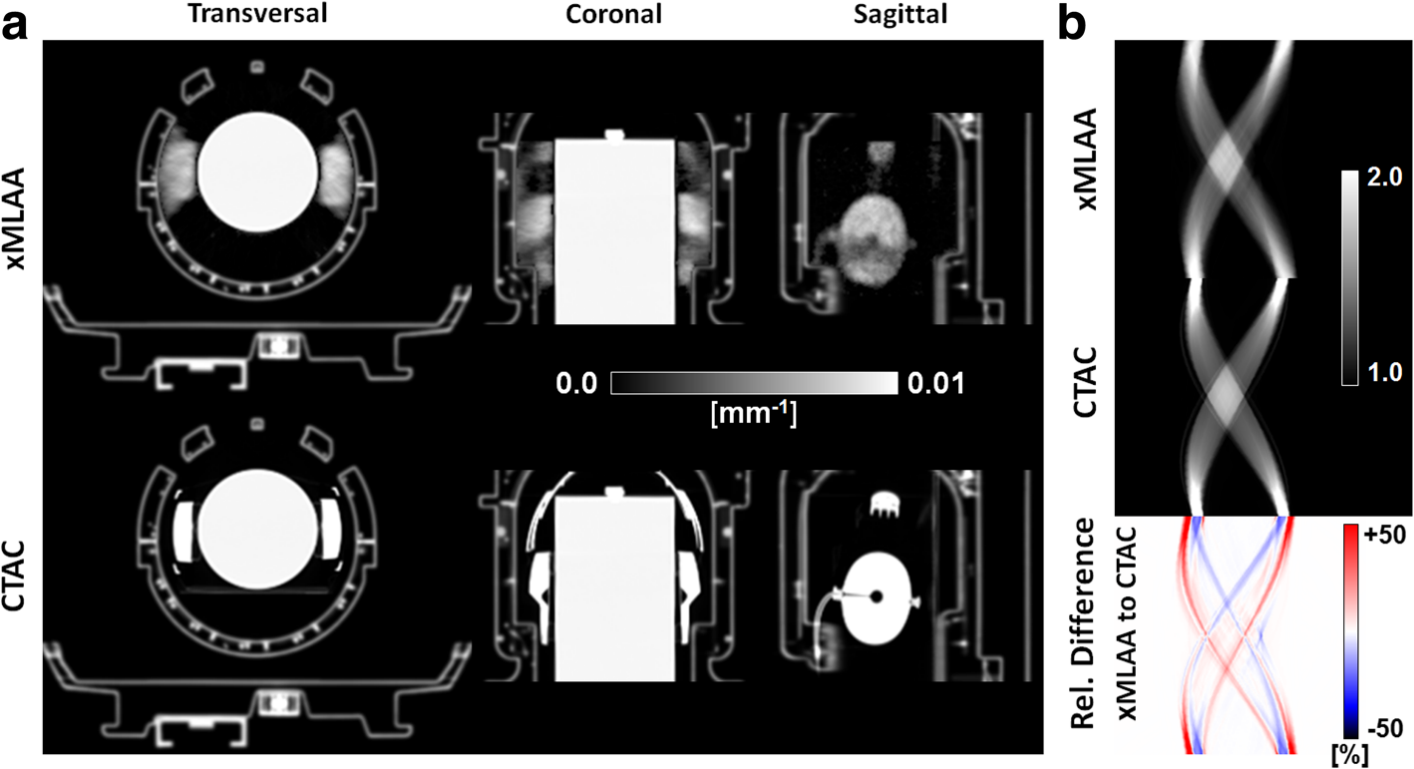
Results for the HP experiment: attenuation. **a** Transversal, coronal, and sagittal views of the xMLAA- and CT-based attenuation maps. **b** Attenuation correction factors (ACFs) of the headphones only. The sinograms correspond to the same transversal plane as shown in (**a**)

Figure 6 presents the xMLAA-obtained attenuation maps in comparison with the CT-based attenuation templates of the headphones. Visual inspection reveals that the xMLAA-derived headphones can be clearly identified and are located in the correct position. A quantitative comparison between xMLAA and CTAC, however, was difficult, because xMLAA was not able to recover the true, physical shape of the headphones, e.g., the earpads as seen in the xMLAA images are wider than in the CTAC images. This observation is in accordance with the results of the simulation study. For a quantitative comparison of the estimated attenuation, we calculated the ACFs of the segmented headphones using Eq. (4). The ACFs are in the same resolution as the emission data, i.e., they were computed along the exact same LORs which were used to model the data acquisition process given by Eq. (3). Sinograms containing the xMLAA- and CT-based ACFs for a direct plane through the center of the PET detector are shown in part (b) of Fig. 6. They show that the ACFs can be accurately estimated for most LORs employing xMLAA, although the difference image reveals a small shift between xMLAA and CTAC, which may be due to slightly inaccurate registration or a slightly different positioning of the headphones during the CT scan. ACFs evaluated within the relevant sinogram space are given in Table 2. The relevant sinogram space is defined by those LORs which contain non-zero values following a forward projection of the hardware mask. Although the maximum ACF is much lower in case of xMLAA compared to CTAC, the average ACF across all evaluated LORs is very similar, indicating that the overall headphone attenuation is accurately estimated by xMLAA.

**Table 2:** Results for the ACFs of the estimated hardware components only, evaluated in the sinogram space which corresponds to the forward projection of the hardware mask. SD specifies the standard deviation. In the uncorrected case, no information on the hardware is available, thus hardware ACFs are always one

	Headphones				RF coil			
	Mean	SD	Min	Max	Mean	SD	Min	Max
Uncorrected	1.000	0.000	1.000	1.000	1.000	0.000	1.000	1.000
xMLAA	1.104	0.171	1.000	2.273	1.065	0.095	1.000	1.894
CTAC	1.107	0.211	1.000	3.541	1.071	0.140	1.000	6.603

**Fig. 7:**
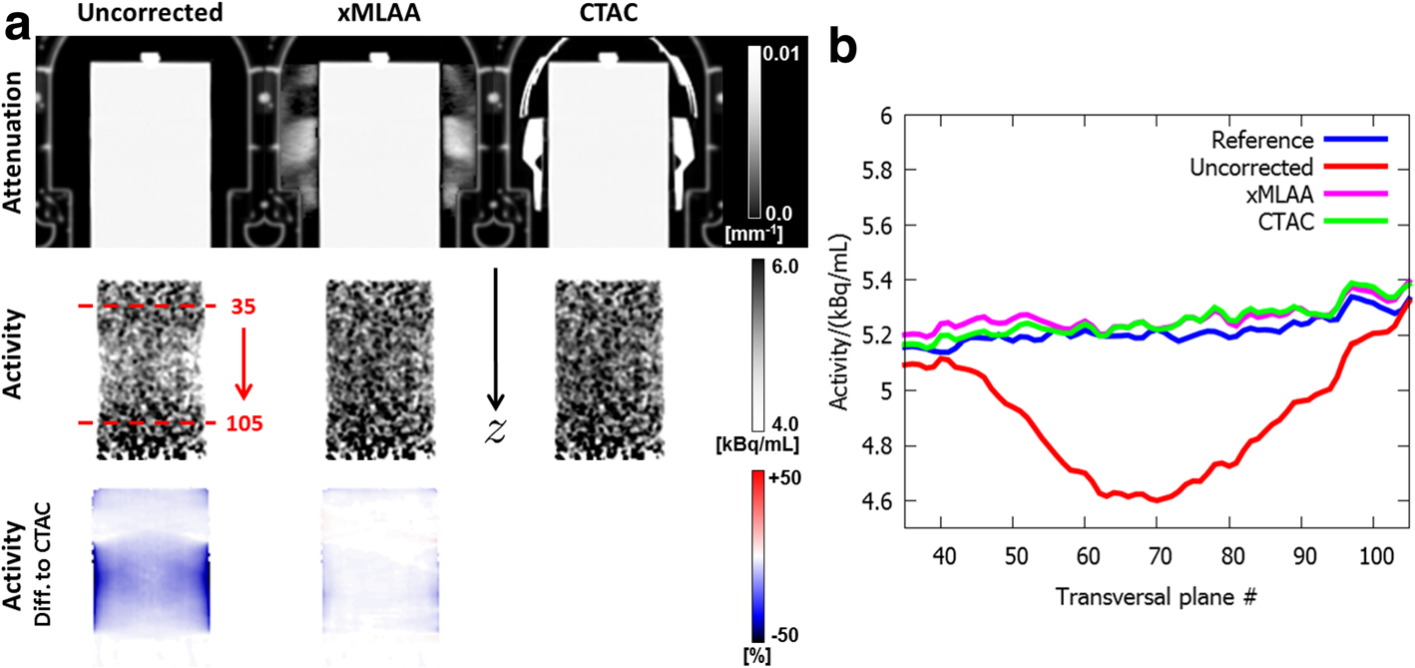
Results for HP experiment: activity. **a** Coronal views of attenuation maps and corresponding activity distributions. Neglecting headphone attenuation (uncorrected) results in an activity underestimation compared to the CT-based reconstruction (CTAC) for regions embraced by the headphones. Correction for headphone attenuation using xMLAA compensates for the activity underestimation. The activity difference images in the *bottom row* give the relative difference to the CT-based reconstruction. **b** Average activity values evaluated within the phantom for transversal planes with varying *z*-position. Plotted are the transversal planes with plane numbers 35 to 105, as indicated in (**a**). In the uncorrected case, the average activity is underestimated by up to 13.4*%* compared to the reference scan. Average activity obtained by xMLAA is slightly higher than in the reference scan with a maximum overestimation of 1.7*%*

The effect of the different attenuation maps on the activity distribution is presented by Fig. 7. For the uncorrected case, i.e., when the headphones were attached to the phantom but neglected during AC, the reconstructed activity was underestimated compared to CTAC, especially for transversal planes embraced by the headphones. The activity underestimation could almost entirely be compensated for when using the xMLAA-obtained attenuation map including an estimation of the headphone attenuation. This becomes most apparent when regarding part (b) in Fig. 7, showing a plane-by-plane evaluation of average activity values within the phantom for transversal planes with varying *z*-position. Compared to the reference scan, the maximum activity underestimation evaluated within the phantom across an entire transversal plane was 13.4*%* when neglecting headphone attenuation (uncorrected). Compensation for headphone attenuation using xMLAA resulted in an activity distribution almost identical to the one obtained using CTAC, with activity differences below 1% for all transversal planes. Compared to the reference scan (reconstructed activity distribution not shown), both xMLAA and CTAC slightly overestimate the activity, with a maximum overestimation of 1.7*%* for xMLAA and of 1.6*%* for CTAC.

#### RF experiment

**Fig. 8:**
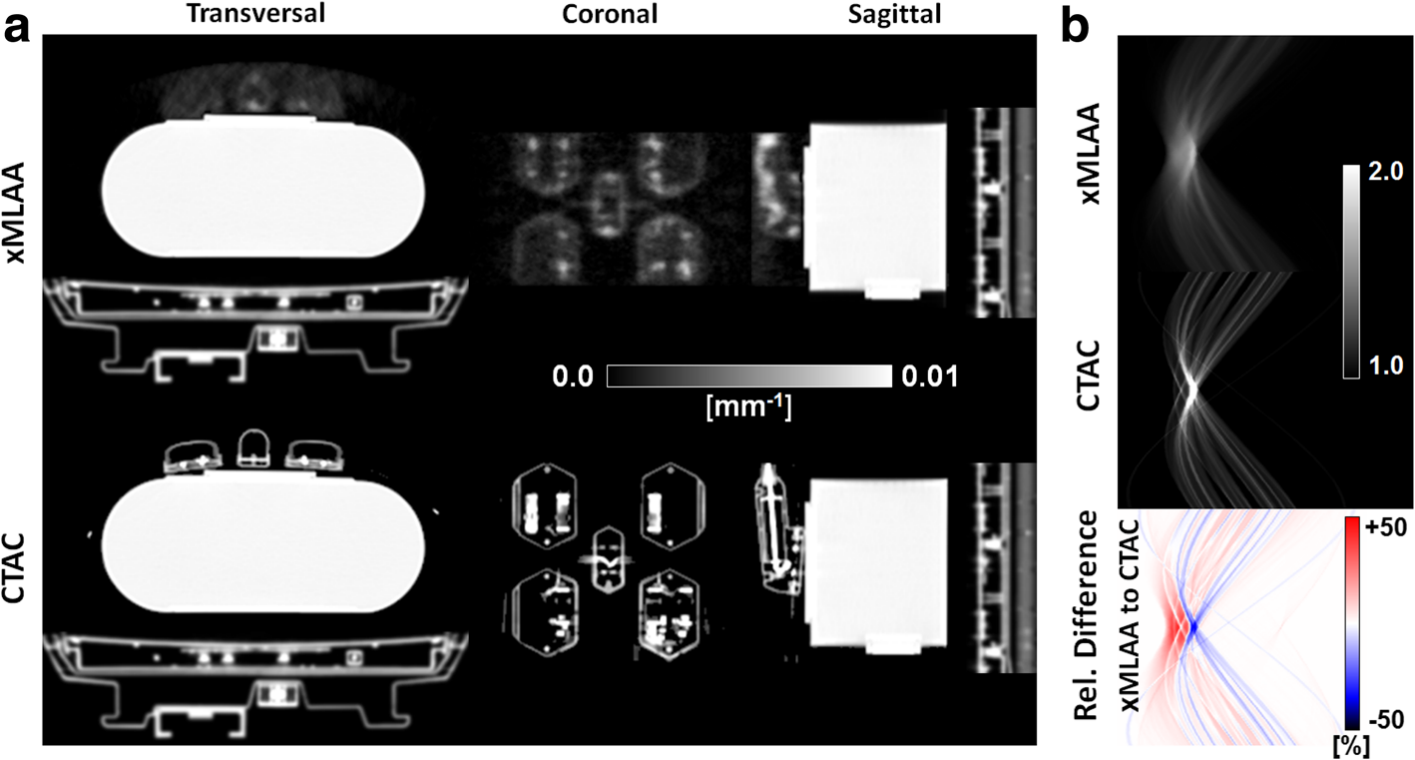
Results for the RF experiment: attenuation. **a** Transversal, coronal, and sagittal views of the xMLAA- and CT-based attenuation maps. **b** Attenuation correction factors (ACFs) of the RF coil only. The sinograms correspond to the same transversal plane as shown in (**a**)

A qualitative comparison of the xMLAA- and CT-based attenuation maps is found in Fig. 8. The different structures of the RF coil visible in the CT-based attenuation map can also be identified when using xMLAA. However, the xMLAA-based attenuation map is impaired by noise and the attenuation coefficients of the different coil structures are lower in general. For quantitative comparison, we calculated the ACFs of the RF coil, as obtained by xMLAA and CTAC. Representative sinograms illustrating the ACFs for a direct plane through the center of the PET detector are given in the part (b) of Fig. 8. In case of xMLAA, the ACFs are much smoother compared to CTAC and the contributions of different coil structures cannot easily be distinguished. As given in Table 2, maximum CT-based ACFs are about 3.5 times higher compared to xMLAA. However, average ACFs evaluated within the relevant sinogram space given by the forward projected hardware mask are very similar.

**Fig. 9:**
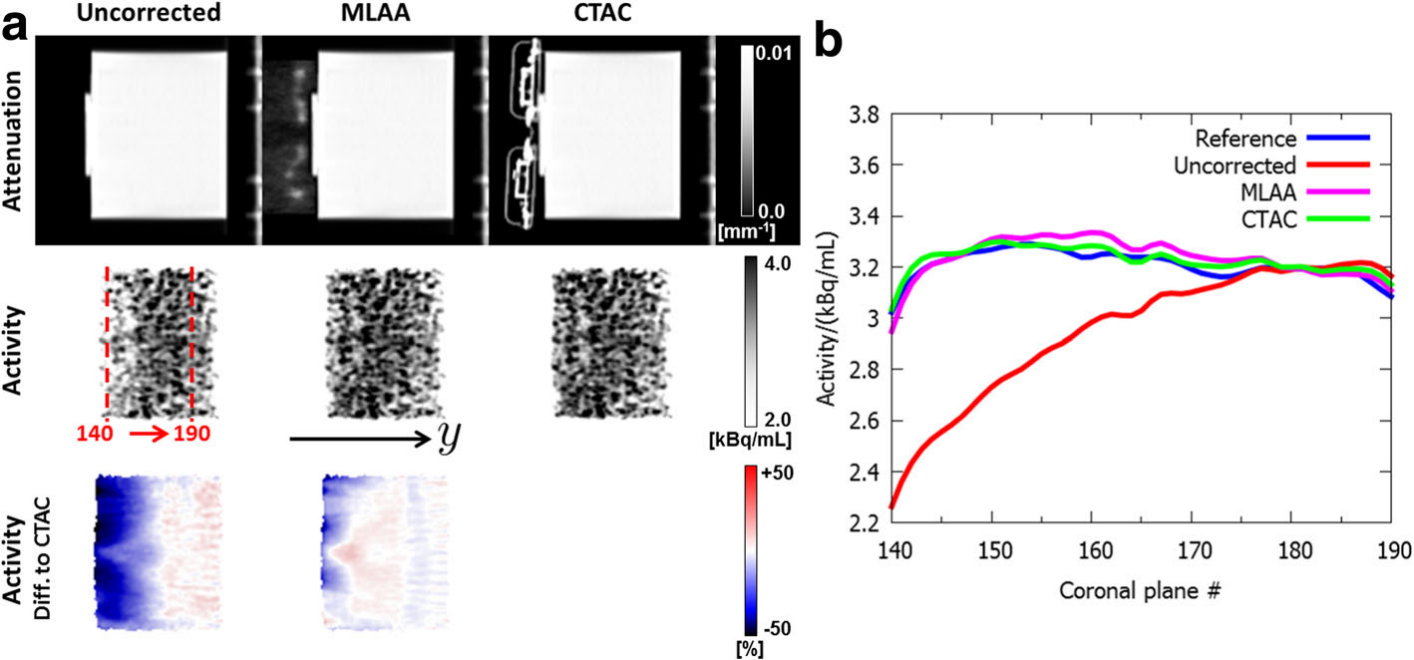
Results for RF experiment: activity. **a** Sagittal views of attenuation maps and corresponding activity distributions. Neglecting headphone attenuation (uncorrected) results in an activity underestimation compared to the CT-based reconstruction (CTAC) for regions in close vicinity of the attached RF coil. Correction for RF coil attenuation using xMLAA compensates for the activity underestimation. The activity difference images in the *bottom row* give the relative difference to the CT-based reconstruction. **b** Average activity values evaluated within the phantom for coronal planes with varying *y*-position. Plotted are the coronal planes with plane numbers 140 to 190, as indicated in (**a**). The activity underestimation present when neglecting headphone attenuation (uncorrected) decreases with increasing distance from the coil. xMLAA results in a slight overestimation of below 1.5*%* for all coronal planes

Figure 9 shows the effect of the different attenuation maps on the reconstructed activity distribution. When ignoring RF coil attenuation (uncorrected), the activity is underestimated, especially for regions in vicinity of the RF coil. The activity underestimation decreases with increasing distance from the coil. For the coronal plane closest to the RF coil, the average activity was underestimated by 25.3*%* compared to the reference scan. The average activity underestimation across all evaluated coronal planes was found to be 8.1*%*. In contrast, compensation for RF coil attenuation employing xMLAA resulted in a slight activity overestimation, which was found to be 0.8*%* on average across all coronal planes. Maximum activity overestimation for a single coronal plane caused by xMLAA was 2.8*%*. Differences between xMLAA and CTAC were below 1.5*%* for all coronal planes.

#### Effect of MRAC-based errors

**Fig. 10:**
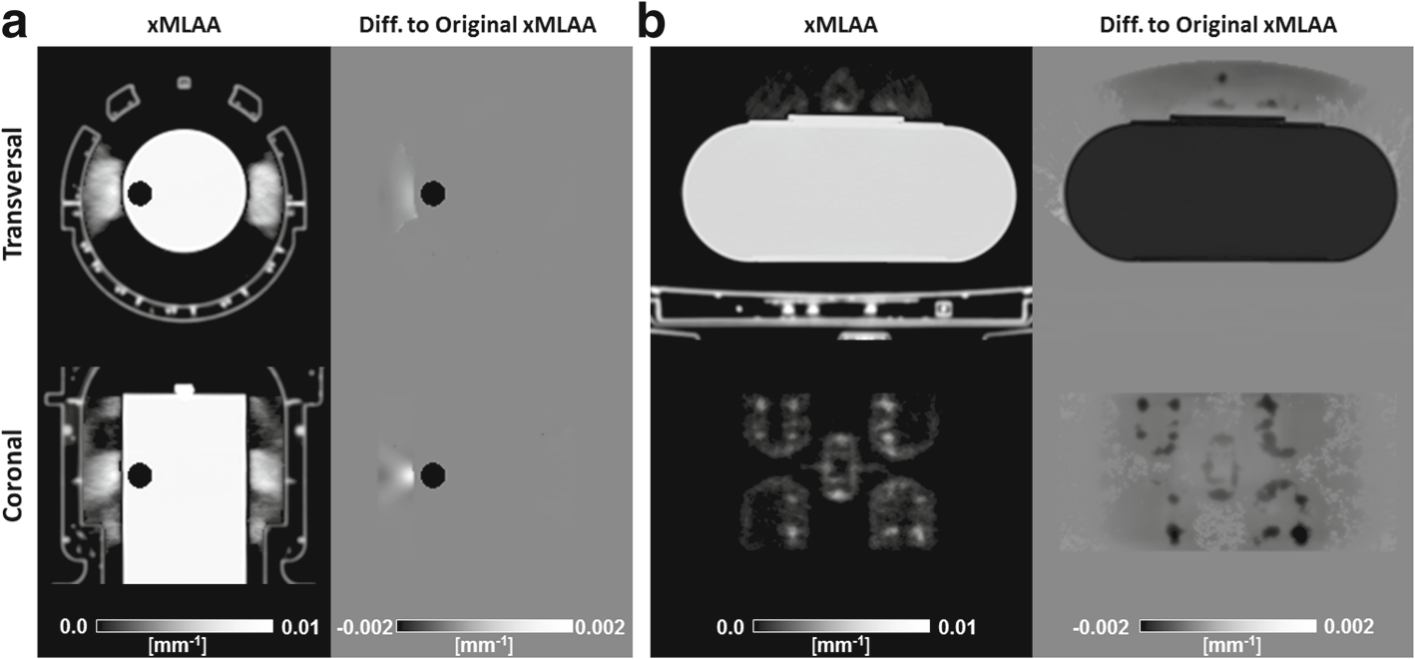
Effect of MRAC-based errors. **a** Transversal and coronal views of the xMLAA-derived attenuation map for the HP experiment in the presence of an intentionally inserted air cavity in close vicinity of one of the earpads. Note, the air cavity is only present in the attenuation map and not in the phantom used during data acquisition. The right column gives the absolute difference to the original xMLAA-based attenuation map, obtained without insertion of the air cavity and shown in Fig. 6. **b** Transversal and coronal views of the xMLAA-derived attenuation map for the RF experiment after an intentional fat-water inversion. The *right column* gives the absolute difference to the original xMLAA-based attenuation map, obtained without fat-water inversion and shown in Fig. 8

The effect of typical errors in the MR-derived attenuation maps on xMLAA-based hardware attenuation estimation is illustrated in Fig. 10. Visual inspection does not reveal any differences compared to the original xMLAA-derived attenuation maps without artifacts, given in Figs. 6 and 8, respectively. Only when considering the corresponding difference images (note the narrow grayscale windowing), the effect of inaccurate attenuation maps on xMLAA-based hardware attenuation can be appreciated. The air cavity inserted to the attenuation map in case of the HP experiment has only a very local effect, while the fat-water inversion in case of the RF experiment reduces the estimated coil attenuation values within the entire hardware mask.

To quantify and compare the direct and the indirect impact of the inaccurate phantom attenuation maps on PET quantification, we evaluated the average activity values throughout the entire phantoms. PET images were reconstructed with the inaccurate phantom attenuation maps and corresponding inaccurate hardware estimates as well as with the original CTAC-derived phantom attenuation maps and the inaccurate hardware estimates. The former demonstrates the direct impact of inaccurate attenuation maps and the latter the indirect impact due to the inaccurate hardware estimation. In case of the artificial air cavity simulated in case of the HP experiment, the direct impact caused an average activity underestimation of 0.4*%* compared to the original xMLAA, while the indirect impact only resulted in an activity overestimation of below 0.1*%*. For the fat-water inversion in case of the RF experiment, the effects were significantly larger, with an activity underestimation of 27.4*%* due to the direct impact and of 1.9*%* due to the indirect impact.

#### Effect of inaccurate scatter estimates

**Fig. 11:**
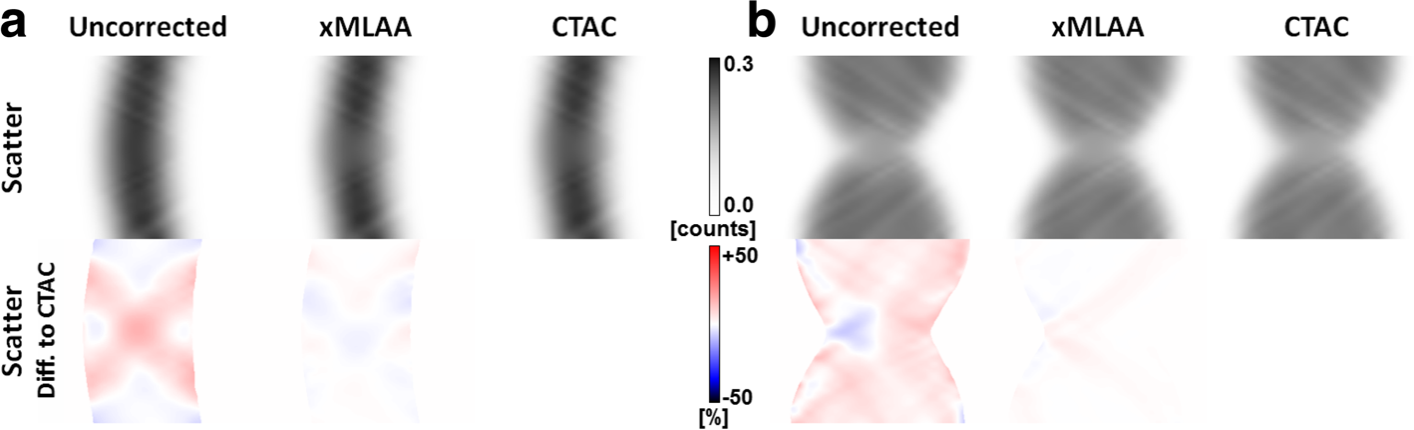
Effects of inaccurate scatter. **a** Sinograms showing the scatter estimates obtained with the SSS algorithm and corresponding to the uncorrected, xMLAA-based, and CTAC-based attenuation maps in case of the HP experiment. The *bottom row* gives the relative difference to the CTAC-based scatter estimate. **b** Sinograms showing the scatter estimates obtained with the SSS algorithm and corresponding to the uncorrected, xMLAA-based, and CTAC-based attenuation maps in case of the RF experiment. The bottom row gives the relative difference to the CTAC-based scatter estimate

The scatter estimates obtained with the uncorrected, xMLAA-based, and CTAC-based attenuation maps are presented in Fig. 11, showing sinograms corresponding to a central direct plane. In the uncorrected case, where the hardware is not considered, scatter is overestimated compared to CTAC, with an average error of +4.0*%* in case of the HP experiment and of +4.5*%* in case of the RF experiment, evaluated in the entire sinogram space. The scatter estimate based on the xMLAA-derived attenuation map is almost identical to the one obtained using the CTAC-based attenuation map, with average errors of only −0.2*%* and +0.9*%* in case of HP and RF experiments, respectively. The inaccurate scatter estimates based on the uncorrected attenuation maps translate into PET quantification errors, which were found to be −1.0*%* and −2.2*%* for the average activity values throughout the entire phantoms in case of HP and RF experiments, respectively. These errors could be reduced to +0.05*%* for the HP experiment and to −0.3*%* for the RF experiments, when comparing the effect of xMLAA-based with CTAC-based scatter estimates.

### Patient data

Since neither a reference scan nor appropriate CT-based attenuation templates were available for the patient data, a thorough quantitative evaluation was not possible. We rather evaluated the effect of ignoring attenuation of flexible hardware components compared to including xMLAA-based attenuation estimates of headphones and RF coils and compared the corresponding observations to the results obtained with the phantom data.

#### HP patients

**Fig. 12:**
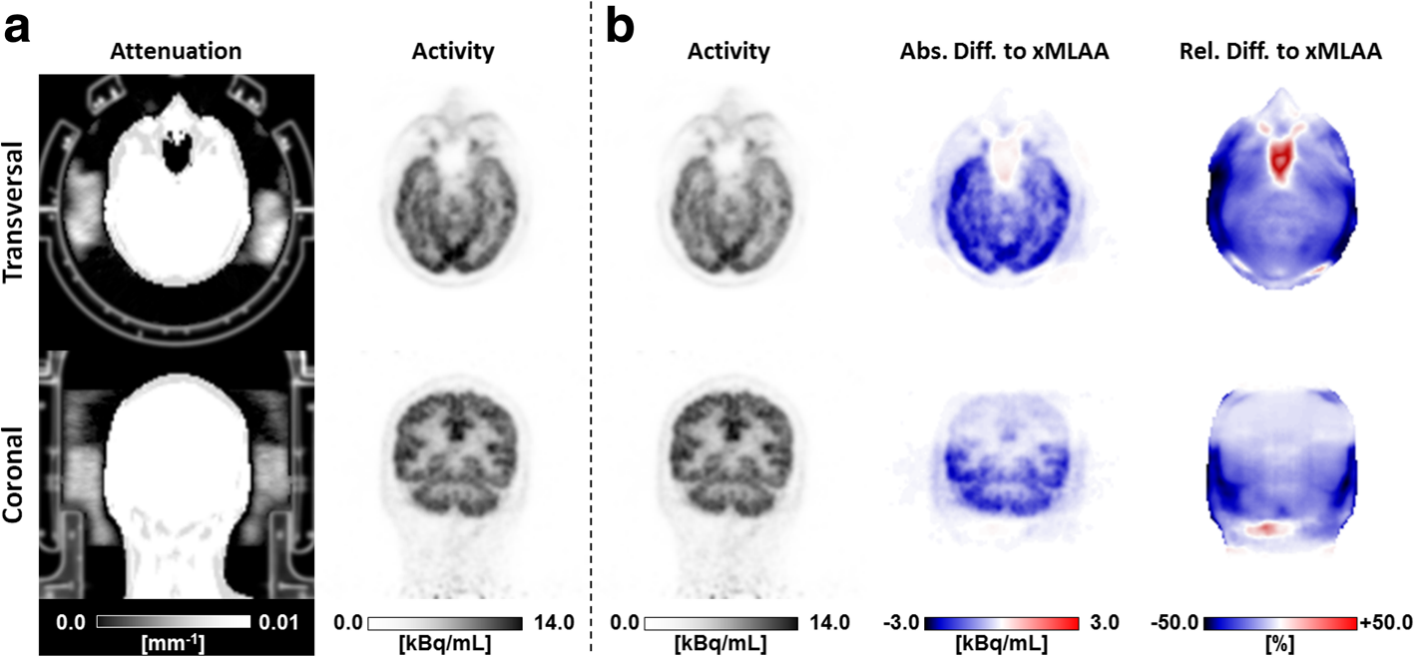
Results for the HP patient data. Transversal and coronal views of an ^18^F-FDG patient wearing headphones. **a** xMLAA-based attenuation map and corresponding activity distribution. **b** Activity distribution obtained when ignoring headphone attenuation (uncorrected) and absolute (Abs) and relative (Rel) difference images to the xMLAA-based activity distribution shown in part (**a**)

Part (a) of Fig. 12 shows the xMLAA-based attenuation map and the corresponding activity distribution for one representative patient included in the study. In the attenuation map, the earpads as well as the headband can clearly be identified. Comparing the xMLAA-based activity distribution with the activity distribution obtained when ignoring headphone attenuation (uncorrected), a severe activity underestimation for the regions embraced by the headphones is found. This becomes most apparent when regarding both the absolute and relative difference images given in part (b) of Fig. 12. Quantitative evaluation across all six patients revealed an average activity underestimation in the full brain of 7.9±0.9*%* compared to xMLAA when ignoring headphone attenuation. Regional activity underestimation, e.g., in the cerebellum, was found to be as large as 13.3±1.2*%* on average across all patients.

#### RF patients

**Fig. 13:**
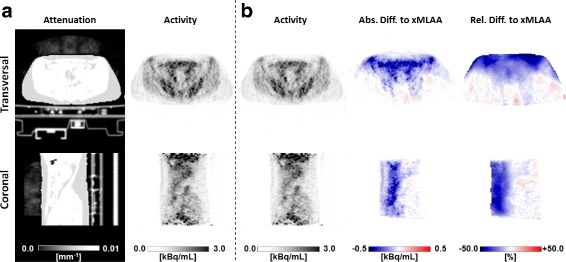
Results for the RF patient data. Transversal and coronal views of an ^18^F-FDG patient with RF surface coils attached. **a** xMLAA-based attenuation map and corresponding activity distribution. **b** Activity distribution obtained when ignoring RF coil attenuation (uncorrected) and absolute (Abs) and relative (Rel) difference images to the xMLAA-based activity distribution shown in part (**a**)

The xMLAA-based attenuation map and the corresponding activity difference images are shown in part (a) of Fig. 13. Compared to the results obtained using the phantom data, the coil attenuation map appears to be more noisy. However, the general structure of the coil can still be identified. Across all five patient data sets and evaluated in the entire torso corresponding to the given bed position, neglecting RF coil attenuation resulted in an average activity underestimation of 6.1±0.9*%* compared to xMLAA. As for the phantom data, activity underestimation is more severe for regions close to the attached RF coil, which is clearly visible in the activity difference images in part (b) of Fig. 13. Regional activity underestimation when neglecting RF coil attenuation was observed to be as large as 19.6*%* compared to xMLAA.

## Discussion

Neglecting attenuation of flexible hardware components, such as MR-safe headphones and RF surface coils, leads to severe activity underestimation, as demonstrated in the work presented here and as reported previously [22, 23, 25, 33, 34, 38]. With improved MR-based patient attenuation correction methods approaching CT-like attenuation maps [12–14], the lack of proper compensation for attenuation of flexible hardware components comes into focus. In fact, for tumors located in close vicinity of one of the earpads of the headphones or in vicinity of highly attenuating parts of the RF coil, neglecting hardware attenuation may easily become the dominating source of error in the reconstructed PET images. In this work, we demonstrated that emission-based estimation of flexible hardware attenuation employing the proposed xMLAA algorithm can be used to significantly improve PET quantification in the affected regions.

As seen from the results presented in Figs. 5, 6, and 8, xMLAA is not able to recover the exact physical shape of the hardware components present in the PET FOV. This is especially apparent for complex and very inhomogeneous objects such as the RF coil. Although the main structures and highly attenuating components can be identified, these structures seem to be blurred and the attenuation coefficients are too low in general. In contrast, the attenuation coefficients in regions which correspond to air are non-zero and thus too high. Even for compact and (almost) homogeneous objects such as the headphones, xMLAA cannot retrieve the exact shape. Moreover, it is obvious that the shape of the estimated hardware components depends on the region where xMLAA is applied, i.e., on the hardware mask illustrated in Fig. 4. We found that the hardware mask should be chosen such that its outer boundary encloses the true physical shape of the hardware components as tightly as possible. If chosen too small, the integrated attenuation along the individual LORs is underestimated. If chosen too large, noise propagation from the emission data into the attenuation map increases the average attenuation coefficients in regions which are assumed to correspond to air, resulting in an overestimation of the integrated attenuation along the individual LORs.

The reason why the exact shape of the hardware components to be estimated cannot be recovered by xMLAA is because the emission-based information on the hardware attenuation is available from a limited number of view angles only, as illustrated in Fig. 5b. Additional prior information, e.g., on the shape and approximate location of the hardware components, will most likely improve the results. Moreover, dedicated limited angle reconstruction techniques for transmission tomography, e.g., employing total variation [54], may be beneficial. While cross-talk between attenuation and activity is a severe problem when applying MLAA for patient attenuation estimation [15], it is not relevant for xMLAA-based hardware estimation as presented in this work. Since the hardware components do not contain activity, consistency conditions provided by LORs crossing the hardware but not the patient force the reconstructed activity to remain close to zero, limiting (direct) cross-talk effects. Therefore, incorporating TOF information is not expected to significantly improve the robustness and accuracy of xMLAA for hardware attenuation estimation. Moreover, restricting the activity update to within the patient body outline, i.e., assuming zero activity outside the patient, was not found to be relevant.

**Fig. 14:**
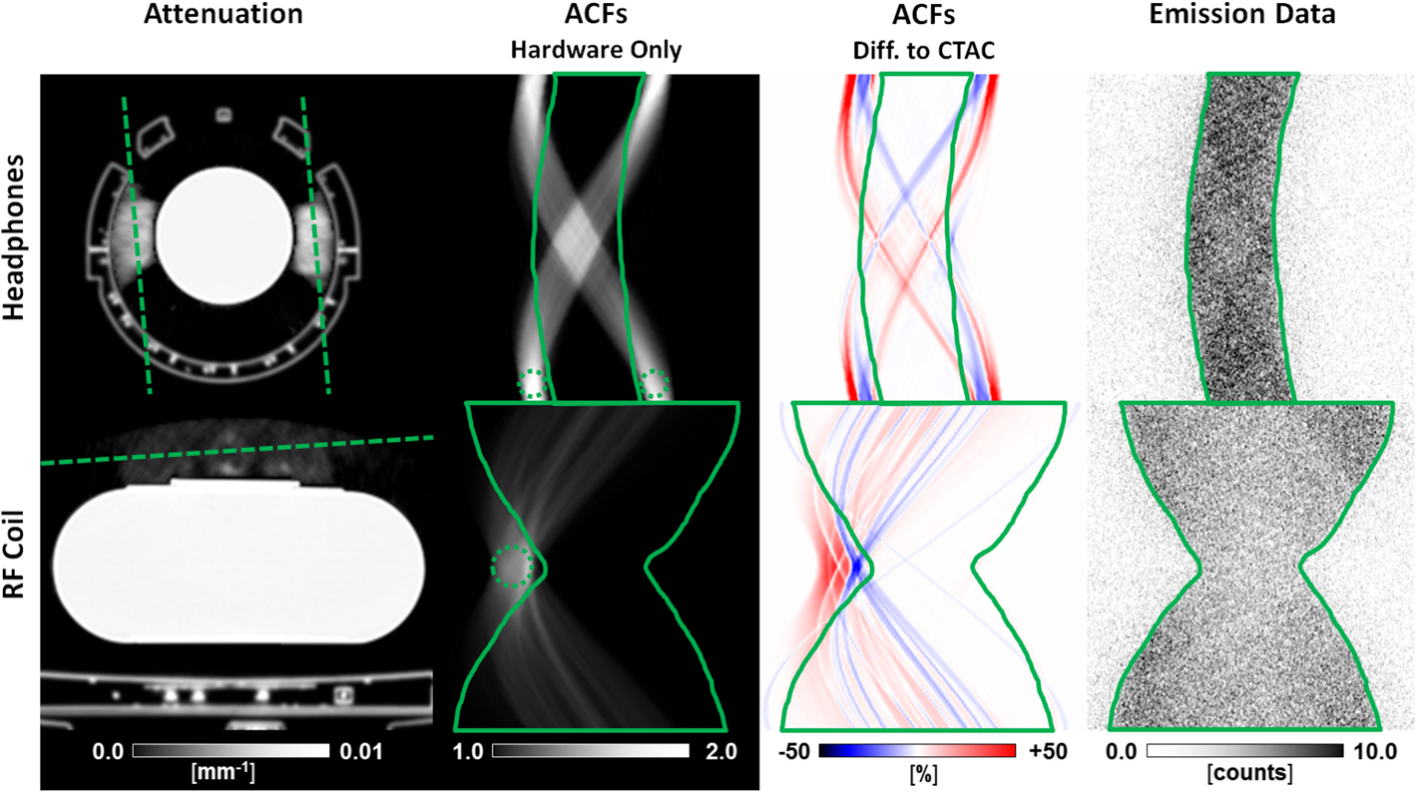
ACFs relevant for PET quantification. Attenuation maps, ACFs, and acquired emission data (after gap filling) in case of headphones and RF coil. The sinograms (ACFs and emission data) correspond to the same transversal plane as used for illustration of the attenuation map. The *solid green line* specifies the manually segmented emission sinogram support. Only LORs located within the emission sinogram support contribute to the PET image of the phantom/patient. This is not the case for the LORs illustrated by the *dashed green lines*, which penetrate only the hardware but not the phantom/patient. Their approximate location within the sinogram space is given by the *dashed green circles*

As demonstrated by the simulation study and the phantom experiments presented in this work and summarized by Table 2, xMLAA is capable to accurately recover the overall hardware attenuation and to obtain estimates for the ACFs of the hardware components which significantly improve PET quantification. Using phantom measurements, it was shown that quantification errors in the reconstructed PET images could be reduced from up to 25% when neglecting hardware attenuation to below 3% when employing xMLAA. The accuracy of the xMLAA-based PET quantification seems surprising, considering that the ACFs obtained with xMLAA may be over- or underestimated by up to ±50*%* for some LORs, as shown in Figs. 6b and 8b for headphones and RF coil, respectively. A closer look at the ACFs is given in Fig. 14. Accurate ACFs are only required for the LORs within the emission sinogram support, defined by the solid green line. All other LORs do not penetrate the phantom/patient and the respective ACFs are thus not needed for accurate PET quantification. As Fig. 14 shows, the errors of the ACFs estimated with xMLAA are quite small for the LORs within the emission sinogram support. Large errors with ±20*%* and more for a single LOR are only observed outside the emission sinogram support. This is reasonable, since for LORs outside the emission sinogram support, e.g., the ones indicated by the dashed green lines, no emission-based information for accurate attenuation estimation employing xMLAA is available. Luckily, and as stated previously, this information is not required for accurate quantification of the reconstructed phantom/patient activity distribution.

It should be noted that proper weighting of the prior terms used in the objective function (1) is crucial for accurate xMLAA-based hardware attenuation estimation. If the weighting of the smoothing and intensity prior is too low, noise propagation from the emission data into the estimated attenuation map causes non-zero average attenuation coefficients in regions which are assumed to correspond to air, i.e., where there should be zero attenuation. On the other hand, if the prior is too strong, the algorithm gets trapped in the local solution defined by the initial attenuation map, i.e., with only air outside the patient outline. It should be noted that proper weighting of the prior terms used in the objective function (1) is crucial for accurate xMLAA-based hardware attenuation estimation. If the smoothing and the intensity prior are not considered or if the weighting of the prior terms is too low, noise propagation from the emission data into the estimated attenuation map causes non-zero attenuation coefficients in regions which correspond to air, i.e., where there should be zero attenuation. On the other hand, if the prior is too strong, the algorithm gets trapped in the local solution defined by the initial attenuation map, i.e., with only air outside the patient outline. In addition, the choice of the pre-defined mean attenuation coefficients and their respective distributions should be reasonable. That is, the expected attenuation coefficient of air should be set to zero with small standard deviation only. For the hardware mean attenuation coefficient, we found that *μ*_hardware_=0.01 mm^−1^ is a good choice both for the headphones and for the RF coil. However, similar results were obtained for all mean values from *μ*_hardware_=0.005^−1^ to 0.015 mm^−1^, as long as the corresponding standard deviation is large enough to allow for a wide range of attenuation coefficients in the reconstructed attenuation maps. Since only the ACFs are relevant for PET quantification, and the shape of the estimated attenuation maps are not important, algorithms directly estimating the ACFs from the emission data such as MLACF [55, 56] may be employed. However, the limited angle problem remains and incorporation of prior information, e.g., using pre-defined attenuation coefficients, is more difficult. Moreover, the concept of applying the attenuation update only within the hardware mask cannot readily be translated to sinogram space.

Inaccuracies in the MRAC-based patient attenuation maps were found to have only minor effects on xMLAA-based hardware attenuation estimation. The corresponding indirect impact of the slightly inaccurate hardware attenuation estimates on PET quantification could be neglected compared to the direct impact of inaccurate patient attenuation maps. We did not evaluate the effect of truncated attenuation maps on xMLAA-based hardware attenuation. However, considering the results presented in Fig. 10 and especially the observation that errors in the patient attenuation map have only local effects on the estimated hardware attenuation, truncated attenuation maps are not expected to significantly influence xMLAA-based hardware attenuation. Similarly, inaccuracies in the scatter estimates, e.g., obtained by neglecting the flexible hardware components during scatter estimation, were not found to have a significant effect on PET quantification. However, we showed that the xMLAA-based scatter estimates were much more accurate than the scatter estimates obtained without considering hardware components (Fig. 11). For best results, scatter estimation would have to be included into the iterative xMLAA hardware attenuation estimation procedure, as already suggested in reference [40].

In the work presented here, quantitative evaluation of the proposed method to estimate attenuation of flexible hardware components was performed for phantom measurements only. We additionally demonstrated the applicability of the proposed xMLAA-based approach to clinical patient data. Although reference scans or aligned CT-based attenuation templates were not available for quantitative evaluation in case of patient data, we observed activity underestimation values in the same range as for the phantom data. These observations suggest that xMLAA can be used to accurately compensate for attenuation of flexible hardware components in clinical patient data. For further investigation, patient studies comparing the proposed approach with registration-based approaches [22, 25, 27, 28, 33] are mandatory. Moreover, an extension to include PET data from several bed positions is required, especially for RF coil attenuation estimation. Finally, the feasibility of the proposed emission-based hardware attenuation estimation needs to be investigated for tracers other than ^18^F-FDG. It is expected that the proposed method works well for tracers with high uptake and a rather homogeneous activity distribution.

## Conclusions

In this work, we proposed to employ a modified MLAA algorithm called xMLAA to estimate the attenuation of flexible hardware components routinely used in hybrid PET/MR imaging. Our results obtained performing dedicated phantom experiments revealed that local errors in the activity distribution when neglecting flexible hardware components could be reduced from up to 25 to below 3% compared to reference scans without hardware present or to CT-based AC. We also demonstrated the feasibility of applying xMLAA-based hardware attenuation estimation to clinical PET/MR data. Since MLAA is already used in clinical routine to extend truncated MR-based attenuation maps, the proposed method can, potentially, be readily included into clinical workflow.
